# Supplementary figures and images for: Production and characterization of antibody against Opisthorchis viverrini via phage display and molecular simulation
Source: PLoS One. 2021 Mar 23;16(3):e0248887. doi: 10.1371/journal.pone.0248887 (PMC7987191; doi:10.1371/journal.pone.0248887)

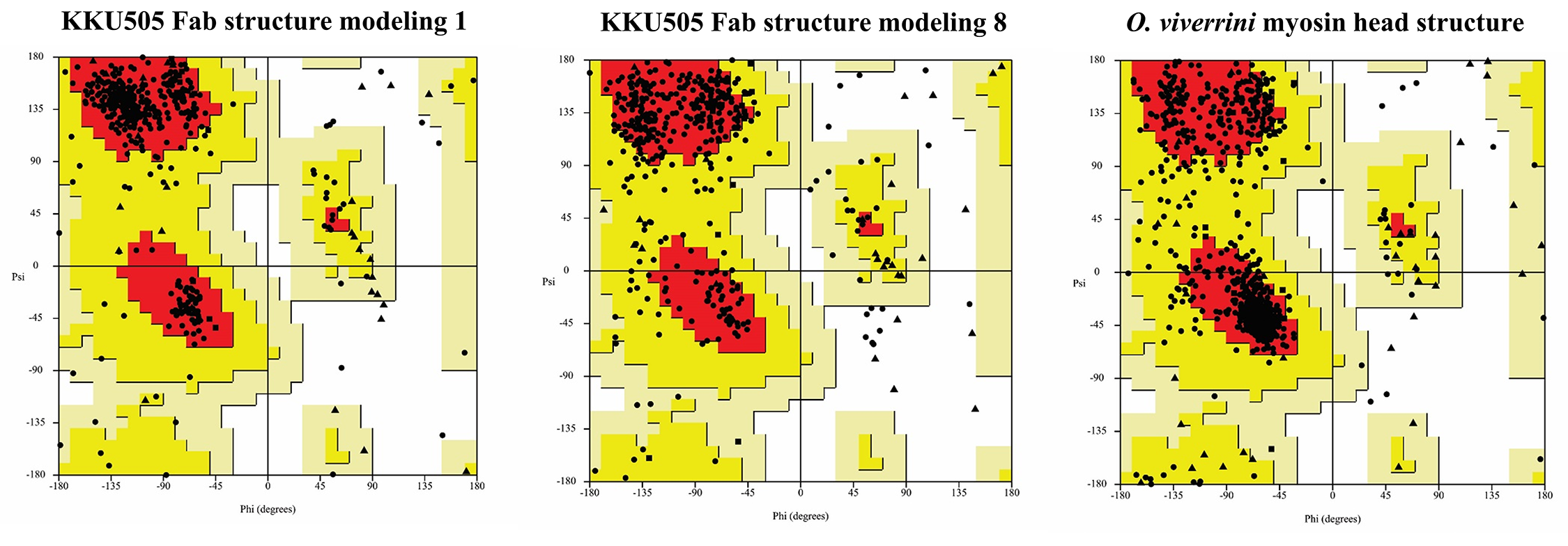

Supplement: S1 Fig — KKU505 Fab and OV myosin head structure structures were validated by Ramachandran plot (red: Favoured region, yellow: Allowed regions, pale yellow: Generously allowed regions and write: Disallow regions). (TIF) [file pone.0248887.s001.tif]

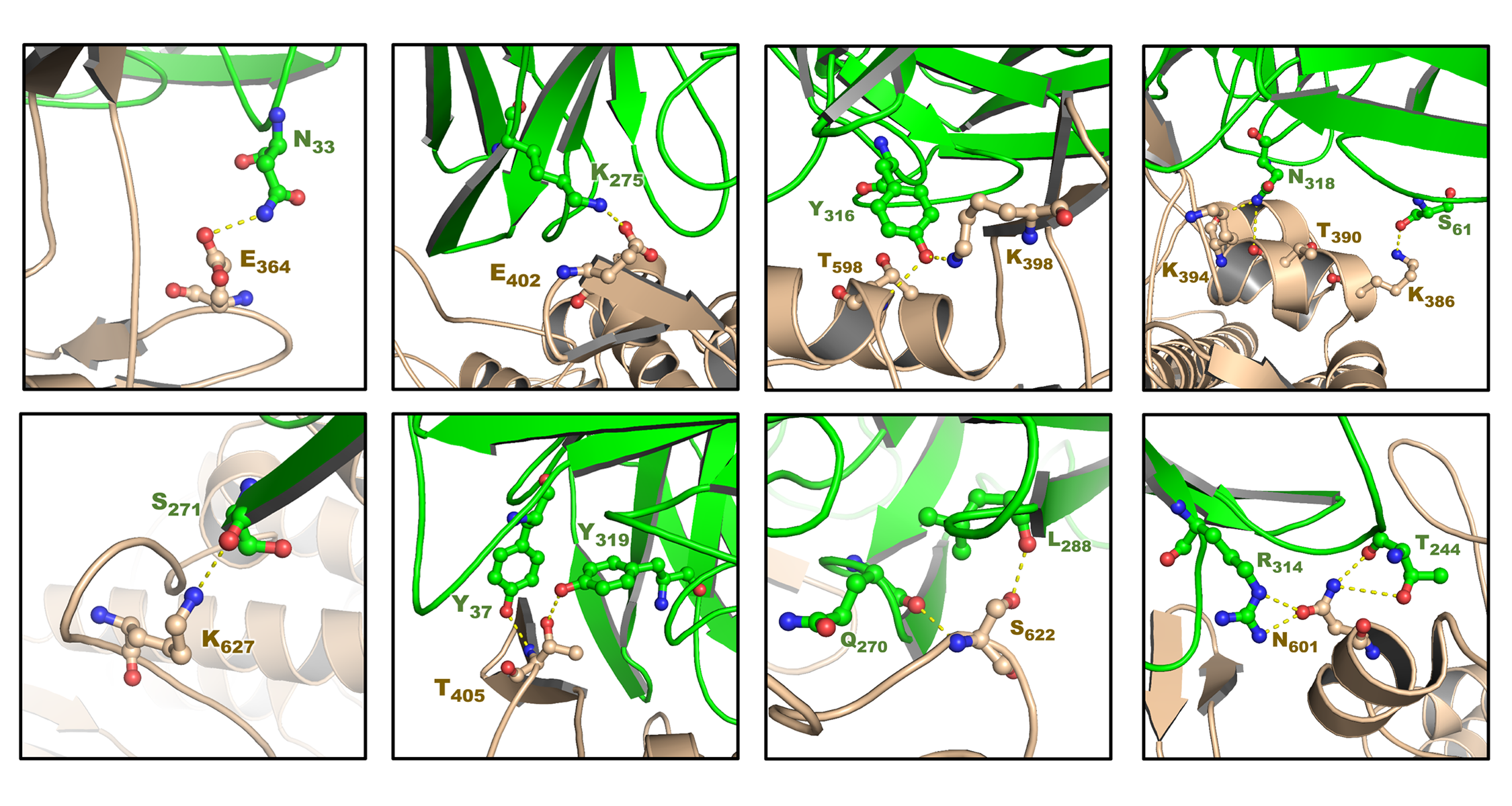

Supplement: S2 Fig — Interactions between KKU505 Fab (green schematic ribbon) and OV myosin head (wheat surface) by ClusPro 2.0. Side-chains are ball and stick. Hydrogen and ionic bonds are dotted lines. Amino acids are abbreviated with a capital. The number is residue position. (TIF) [file pone.0248887.s002.tif]
