# Supplementary material for: Production and characterization of antibody against Opisthorchis viverrini via phage display and molecular simulation
Source: PLoS One. 2021 Mar 23;16(3):e0248887. doi: 10.1371/journal.pone.0248887 (PMC7987191; doi:10.1371/journal.pone.0248887)
Supplement: S1 File — (DOCX) [file pone.0248887.s003.docx]

**Minimal data set**

# Phage ELISA data

For specificity characterization of antigen-specific binding of phage presenting KKU505 Fab fragment (KKU505 phage 1 and 8)

**Normalized absorbance at 450 nm**

| **Antigen** | **With crude OV antigen (1 µg/well)** | | | |
| --- | --- | --- | --- | --- |
| **Phage** | **KKU505 phage 1** | **KKU505 phage 8** | **1H10 MICA Phage** | **VSCM13 helper phage** |
| **1** | 0.663 | 1.046 | 0.145 | 0.057 |
| **2** | 0.663 | 1.166 | 0.151 | 0.049 |
| **3** | 0.683 | 1.127 | 0.154 | 0.051 |
| **Mean** | **0.670** | **1.113** | **0.150** | **0.052** |
| **SD** | **0.012** | **0.061** | **0.005** | **0.004** |
| **P-value** | **<0.0001** | **<0.0001** | **<0.0001** | **<0.0001** |

For sensitivity or concentration limitation, KKU505 phage 8 was performed to characterize the ability of KKU505 phage 8 to determine relation between crude OV antigens concentration and KKU505 phage 8 using indirect ELISA.

**Normalized absorbance at 450 nm**

| **Antigen** | **Crude OV antigen** | | | | | | |
| --- | --- | --- | --- | --- | --- | --- | --- |
|  | **2**  **µg/well** | **1**  **µg/well** | **0.5**  **µg/well** | **0.25**  **µg/well** | **0.1**  **µg/well** | **0.05 µg/well** | **0**  **µg/well** |
| **Phage/ antibody** | **KKU505 Phage 8** | | | | | | |
| 1 | 1.099 | 0.969 | 0.882 | 0.735 | 0.588 | 0.489 | 0 |
| 2 | 1.095 | 1.008 | 0.898 | 0.769 | 0.64 | 0.544 | 0 |
| 3 | 1.039 | 0.959 | 0.88 | 0.687 | 0.566 | 0.476 | 0 |
| **Mean** | **1.078** | **0.979** | **0.887** | **0.730** | **0.598** | **0.503** | **0.000** |
| **SD** | **0.034** | **0.026** | **0.010** | **0.041** | **0.038** | **0.036** | **0.000** |
| **P-value** | **<0.0001** | **<0.0001** | **<0.0001** | **<0.0001** | **<0.0001** | **<0.0001** | **ND** |

| **Antigen** | **BSA** | | | | | | | |
| --- | --- | --- | --- | --- | --- | --- | --- | --- |
|  | **2**  **µg/well** | **1**  **µg/well** | **0.5**  **µg/well** | **0.25 µg/well** | **0.1**  **µg/well** | **0.05 µg/well** | **0**  **µg/well** | **0 µg/well** |
| **Phage/ antibody** | **KKU505 Phage 8** | | | | | | | **No Phage** |
| 1 | 0.131 | 0.035 | 0.017 | -0.005 | -0.015 | 0.017 | 0.003 | 0 |
| 2 | 0.144 | 0.061 | 0.052 | 0.033 | -0.014 | -0.012 | 0.006 | 0 |
| 3 | 0.132 | 0.025 | 0.05 | 0 | -0.056 | -0.045 | -0.024 | 0 |
| **Mean** | **0.136** | **0.040** | **0.040** | **0.009** | **-0.028** | **-0.013** | **-0.005** | **0.000** |
| **SD** | **0.007** | **0.019** | **0.020** | **0.021** | **0.024** | **0.031** | **0.017** | **0.000** |
